# 597. Comparing Distraction Methods for Pediatric Pain Management During Burn Dressing Changes

**DOI:** 10.1093/jbcr/irag033.401

**Published:** 2026-04-06

**Authors:** Sarah A Castiel, Emily Snyder, Jennifer D Rosenthal, Devika Pavuluri

**Affiliations:** Medical City Plano, Plano, TX; Medical City Plano, Plano, TX; Medical City Plano, Plano, TX; Medical City Plano, Plano, TX

## Abstract

**Introduction:**

Pediatric burn dressing changes can be extremely painful, causing distress, anxiety and often times can be a traumatizing experience for the patient. Unique, effective, nonpharmacologic methods of pain reduction are needed to augment pharmacologic interventions to help reduce burn treatment pain for our pediatric populations. At our American Burn Association (ABA) verified burn outpatient clinic, a deluxe mobile sensory station (DMSS) was introduced to help transform a treatment room into a relaxing space and provide needed distraction to the pediatric patient. The DMSS is comprised of an interactive bubble tube with color changing effects, a projector which can display a variety of images, and fiber optic tails which display colors and provide soothing pressure. However, there are no studies to the authors’ knowledge showing that the DMSS can also help with pain reduction in pediatric burn patients.

**Methods:**

An IRB approved randomized controlled study was executed to compare pain scores before, during, and after wound care using DMSS distraction versus traditional distraction provided by the CCLS. Eligible patients for the study were pediatric patients ages 3-10 years of age and who were experiencing their initial visit to the outpatient burn clinic. Excluding criteria were patients who were developmentally delayed, neurodivergent, or did not speak English. This study was a randomized control trial where the patients were randomly assigned, by alternating every other patient, the DMSS or traditional child life distraction during their first visit. Nurses were instructed to provide the care they usually provide to any patient receiving wound care in the outpatient clinic. Throughout the dressing change, (before, during and after) the patient was provided with the Wong-Baker FACES pain scale to rate their pain. Parents were asked to rate their child’s pain using the numeric pain scale (NRS). Nurses were asked to rate the patients' pain using the FLACC pain scale.

**Results:**

A Mann–Whitney U, Chi-square analysis, and a regression analysis were completed and revealed no statically significant difference in the patients who received the DMSS or traditional distraction.

**Conclusions:**

There appears to be no significant association between the distraction method (DMSS or Standard) and pain/distress ratings (FACES, FLACC, NRS Parent) at any of the time points (before, during, after the dressing change). However, the lack of statistical significance does not mean that the data collected is not clinically significant. Larger samples sizes may be necessary to see true statistical significance.

**Applicability of Research to Practice:**

As mentioned before, controlling the pain in the pediatric population is complex and requires many different tools and interventions. The DMSS may be able serve as an adjunct tool in making the pediatric patient more comfortable in painful dressing changes.

**Funding for the study:**

N/A.

